# Association study of candidate genes for primary cataracts and fine-mapping of a candidate region on dog chromosome 1 in Entlebucher mountain dogs

**Published:** 2008-05-16

**Authors:** Christina Müller, Ottmar Distl

**Affiliations:** Institute for Animal Breeding and Genetics, University of Veterinary Medicine Hannover, Foundation, Hannover, Germany

## Abstract

**Purpose:**

The Entlebucher mountain dog (EMD) shows a high incidence of primary non-congenital cataracts (CAT). Because of the late-onset of CAT, it is difficult to exclude affected animals from breeding. A screen of candidate genes should help to identify the genes associated with CAT in EMD.

**Methods:**

We genotyped 39 flanking microsatellite markers for 31 cataract candidate genes in 10 EMD families and tested them for linkage and association. For delimitation of a linked chromosome region on canine chromosome 1 (CFA1), we interrogated CFA1 by genotyping 30 additional microsatellites. We also sequenced the whole coding sequence with flanking intronic and untranslated regions of two candidate genes on CFA1.

**Results:**

We found a genome-wide significant genomic region on CFA1, which showed a significantly associated haplotype with the CAT phenotype in the EMDs. Sequencing two candidate genes located on CFA1 revealed three single nucleotide polymorphisms (SNPs), which were not associated with CAT.

**Conclusions:**

We identified a putative CAT region that peaked at 96.07 Mb with genome-wide significant test statistics and extended over 1.3 Mb on CFA1 in the EMD. A significant marker-trait association based on haplotypes corroborated this CAT region. Further research is necessary to determine the gene responsible for CAT that is harbored by this linked and associated genomic region.

## Introduction

Primary cataract is one of the most frequent genetic eye diseases among purebred dogs. There are more than 120 dog breeds in which cataracts are known or presumed to be hereditary [1-7].

In the Entlebucher mountain dog (EMD), primary non-congenital cataract (CAT) is the most frequently observed hereditary eye disease with a prevalence of 23.5% [8]. Most of the affected dogs develop bilateral symmetric opacifications, which are mostly located capsular and subcapsular in the posterior polar part of the lens along the suture lines [9]. The first signs of CAT can be seen at a mean age of 5.5±2.6 years [8].

When complex segregation analyses were employed for pedigree analyses, a mixed major recessive gene model was found to be the most likely mode of inheritance of CAT in EMD (O. Distl and H. Hamann, unpublished results). The heritability was h^2^=0.32±0.05 in an animal threshold model [8]. The animal model uses all relationships of the pedigree of the dogs with phenotypic records to calculate heritabilities, and the threshold model takes into account the binomial nature of the phenotypic trait.

Because of the late-onset of CAT in this breed, it is difficult to exclude either CAT susceptible affected animals early in life from breeding or to ascertain unaffected carriers. A DNA test, which shows whether the dog is homozygous for a CAT-causing mutation or a heterozygous carrier or free from CAT-causing mutations, would be very helpful. Combined with an adequate breeding program, the prevalence of CAT could be effectively decreased in this breed.

To date, there are more than 30 genes that have to be considered as possible candidate genes for primary cataracts in dogs because these genes were found to be associated with hereditary cataracts in humans or mice [10-14]. These genes encode structural or membrane transport proteins of the lens, transcription factors, which are involved in eye and lens development, or enzymes, which are necessary for lens metabolism [12]. Altogether 28 candidate genes for CAT were reported by Mellersh et al. [14] and Hunter et al. [13]. We investigated these 28 candidate genes, and three further genes were added in our analysis. The objective of the present analysis was to evaluate 31 candidate genes for linkage and association with primary non-congenital cataracts in the purebred dog breed EMD. As a genomic region linked with primary cataracts was identified on CFA1, a mutation analysis of the two positional candidate genes was performed and microsatellites were genotyped to confirm this CAT-linked region.

## Methods

### Animals, phenotypic data, and DNA specimens

The ophthalmologic examinations of the dogs were performed by veterinary specialists of the Dortmunder Kreis, the German panel of the European Eye Scheme, for diagnosis of inherited eye diseases in animals (DOK).

The Schweizer Sennenhund Verein für Deutschland e.V. (SSV; kennel club for Swiss mountain dogs in Germany) provided the ophthalmologic data recorded by the DOK and the pedigree data for the EMDs. The pedigree data were collated with the veterinary records to establish pedigrees with multiple CAT-affected dogs.

For our study, we chose 88 purebred EMDs belonging to 10 families consisting of 36 paternal half-sib and 49 full-sib groups. Full-sibs included between one and six dogs. For these dogs, a blood sample was collected and preserved in EDTA. Of these 88 dogs, 65 exhibited clinical signs of lens opacifications. Fifty-four (61.54%) of these 65 dogs had a posterior polar cataract (CAT) approved by a veterinary ophthalmologist of the DOK. Progressive retina atrophy was not seen in the dogs selected for our analysis. Dogs affected by a posterior polar cataract were treated as affected and dogs free from any signs of lens opacifications as non-affected. All other dogs with a phenotype other than a posterior polar cataract or missing phenotypic records were treated as unknown phenotypes in the analysis. About 70% of the CAT-affected dogs were in paternal half-sib groups with two to five affected half-sibs. We had about 52% of the CAT-affected dogs in full-sib groups with two to three affected full-sibs. CAT was diagnosed at a mean age of 5.46±2.64 years. The mean age of all dogs at the last ophthalmologic examination was 6.00±2.79 years.

We performed a mutation analysis for dogs affected by posterior polar cataract using two candidate genes on CFA1 because their flanking markers cosegregated with the CAT phenotype. For this purpose, we chose 37 animals randomly sampled from the population of EMDs. Of these dogs, 25 were affected by a posterior polar cataract (CAT) and the 12 remaining dogs were unaffected.

Two milliliters of heparinized blood were obtained from each dog, and DNA was extracted using a QIAamp 96 DNA Blood Kit (Qiagen, Hilden, Germany).

For the cDNA analysis of the *LIM2* gene, we used lens tissue of a euthanized Tibetan terrier, which was unaffected by CAT. After removal from the eye, the lens tissue was preserved using RNA-later solution (Qiagen). The RNA was extracted from dog lens tissue using the Nucleospin RNA II-Kit (Macherey-Nagel, Düren, Germany) and transcribed in cDNA using SuperScript III Reverse Transcriptase (Invitrogen, Karlsruhe, Germany).

### Genotyping of microsatellites and single nucleotide polymorphism markers

For the investigation of 20 candidate genes, we used microsatellites and polymerase chain reaction (PCR) conditions according to Mellersh et al. [14]. For the remaining 11 candidate genes, flanking microsatellites were obtained by searching the dog genome assembly 2.1 for known microsatellites with a distance of less than one megabase (Mb) to the particular candidate gene. In total, 39 flanking microsatellite markers were genotyped for 31 cataract candidate genes. For the further analysis of the linked region on canine chromosome 1 (CFA1), we chose 30 additional microsatellite markers from the dog genome assembly 2.1. The PCR primers and conditions are shown in Appendix 1.

The PCR for genotyping the microsatellites started at 94 °C for 4 min followed by 38 cycles at 94 °C for 30 s, at optimum annealing temperature for 1 min, at 72 °C for 30 s, and at 4 °C for 10 min. All PCR reactions were performed in 11.5-µl reactions using 6 pmol of each primer, 0.2 µl dNTPs (50 μM), and 0.1µl TaqDNA polymerase (5 U/µl; Roche, Mannheim, Germany) in the reaction buffer supplied by the manufacturer for 1.5 μl of template DNA. The forward primers were labeled fluorescently with IRD700 or IRD800. For the analysis of the marker genotypes, PCR products were size-fractionated by gel electrophoresis on an automated sequencer (LI-COR, Lincoln, NE) using 4% polyacrylamide denaturing gels (Rotiphorese Gel40, Carl Roth, Karlsruhe, Germany). Allele sizes were detected using an IRD700- and IRD800-labeled DNA ladder. The genotypes were assigned by visual examination.

### Non-parametric linkage analysis and association analysis

A non-parametric multipoint linkage analysis was employed for the EMD families using the MERLIN 1.1.1 software (Center for Statistical Genetics, Ann Arbor, MI) [15]. This multipoint analysis is based on allele sharing among affected individuals by identical-by-descent methods [16]. The approach employed appears useful for traits under complex inheritance models because no assumptions have to be made for the genetic parameters. The Whittemore and Halpern non-parametric linkage (NPL) pairs statistics, Z-mean, and LOD score according to Kong and Cox [16] were used for the multipoint chromosome-wide search for allele sharing among affected family members. In the case of no linkage, the Z-mean approaches the minimum achievable value due to an equal distribution of alleles among affected relatives. When linkage is present under the alternative hypothesis, the proportion of alleles identical-by-descent (IBD) deviates significantly from the expected IBD proportions of the null hypothesis. We employed multipoint analyses to make use of all marker information from CFA1 to linked informative markers and to increase power of linkage analysis. Thus, the test statistics are dependent of the usefulness of the markers, their distance to each other, and the number of markers employed in the analysis. This means that Z-means and LOD scores of the same markers can achieve higher values in multipoint analyses when the information content of the linked haplotype is increased through markers with high information content and in close proximity to the causal gene. In contrast to multipoint analyses, Z-means and LOD scores from two-point analyses are not changing when the number of markers is increased and are independent of neighboring markers. For the genome-wide type-I error probability (p_g_), a Bonferroni correction was applied for the chromosome-wide error probability (p) with p_g_=1-(1-p)^1/r^ where r is the ratio of the length of CFA1 (126 Mb) to the total length of the canine genome with 2425 Mb. We used Bonferroni’s procedure to strictly control the overall type-I error rate of genome-wide error probabilities. Haplotypes were estimated using MERLIN 1.1.1 with the option “best.” A case-control analysis based on χ^2^-tests for genotypes, alleles, and trend of the alleles was also performed for the EMDs. The CASECONTROL, HAPLOTYPE, and ALLELE procedures of SAS/Genetics (SAS Institute Inc, Cary, NC) were used for association tests for single markers, marker-trait association tests for haplotypes, tests for Hardy–Weinberg equilibrium of genotype frequencies, and the estimation of allele frequencies [17]. We screened all possible haplotypes of CFA1, including two to four adjacent markers, for association with the CAT phenotype.

### Mutation analysis of candidate genes

We searched the dog expressed sequence tag (EST) archive for ESTs by cross-species BLAST searches with the corresponding human reference mRNA sequences for *FTL* (NM_000146) and *LIM2* (NM_030657). We found a canine EST (CN000212) isolated from dog brain tissue with 88% identity to the human *FTL* mRNA sequence. A significant match to this canine EST was identified on canine chromosome 1 (NC_006583) by means of BLASTN searches of this canine EST against the dog genome assembly (dog genome assembly 2.1). We also found two canine ESTs (DN868840 and DN864011) isolated from beagle lens tissue, which together cover the human *LIM2* mRNA sequence (NM_030657). Significant matches to these canine ESTs were identified on canine chromosome 1 (NC_006583) of the dog genome assembly (dog genome assembly 2.1). The canine *LIM2* ESTs were verified by sequencing the cDNA of *LIM2* that was isolated from the dog lens tissue. The genomic structures of the canine *FTL* and *LIM2* genes were determined using the Spidey mRNA-to-genomic alignment program. The PCR primers are listed in Table 1. PCR primers were designed using the Primer3 program based on the genomic sequence for canine chromosome 1 (NC_006583) and the canine EST sequences of *FTL* (CN000212) and *LIM2* (DN868840 and DN864011).

**Table 1:** Amplification primers for *FTL* and *LIM2*.

**Gene**	**Target**	**Primer**	**Sequence (5’-3’) of primers**	**T_a_ (°C)**	**Product size (bp)**
FTL	337 bp before start codon, exon 1, and intron 1	FTL_1_F	CAGCTCGGATTGGTCAATAG	58	725
		FTL_1_R	CGCTGGTTTTGCATCTTC		
	Exon 2, intron 2, and exon 3	FTL_2_F	GCAGCCTTTGTCTCGTTG	58	689
		FTL_2_R	AAACCTGCCCGATTAAATTC		
	Intron 3, exon 4, and 315 bp after stop codon	FTL_3_F	GGATCCCCATGTAAGTACCC	58	594
		FTL_3_R	TAGGCCTTCCAGAACAACAG		
LIM2	5’UTR, exon 1, and exon 2	LIM2_1_F	GGAGGCTTAAGGGATTTGG	58	780
		LIM2_1_R	TCATGCCAGGAAATGTCAC		
	Exon 3	LIM2_2_F	CACCCATTAGCAAACCAAAC	58	599
		LIM2_2_R	GAGAAGAAACACCCCAGAAAG		
	Exon 4	LIM2_3_F	TACACAGCACTGGCCTGAC	58	645
		LIM2_3_R	AGTGGCCCCATTCTAACTTC		
	Exon 5 and 3’UTR	LIM2_4_F	TGAGCCAGAAGACAGACTCC	58	667
		LIM2_4_R	AGAATTTGACCTATACATCTGTTTCC		
	cDNA	LIM2_F	AGGCTCCAGTCCCTTCCTC	59	660
		LIM2_R	CTCCCCCTCCTTTTCAGTG		

PCR primers, their product size, and annealing temperature (Ta) for the amplification of the genomic sequence of canine *FTL* and for the amplification of the genomic and cDNA sequence of canine *LIM2* are presented.

We sequenced the complete genomic sequence of the canine *FTL* gene and the exonic sequences with flanking intronic and untranslated regions of the *LIM2* gene for the 37 animals mentioned above. All PCRs were performed in 50-µl reactions using 50 pmol of each primer, 100 μM dNTPs, and 2 U TaqDNA polymerase (Q-BIOgene, Heidelberg, Germany) in the reaction buffer supplied by the manufacturer and 10X PCR Enhancer (Invitrogen) for 2 μl template DNA. The PCR conditions were 95 °C for 4 min followed by 38 cycles of 94 °C for 30 s, annealing temperature of 58 °C for 45 s, 72 °C for 60 s, and 4 °C for 10 min. All PCR products were cleaned using the Nucleo-Fast PCR purification kit (Macherey-Nagel) and directly sequenced with the DYEnamic ET Terminator kit (GE Healthcare, Freiburg, Germany) and a MegaBACE 1000 capillary sequencer (GE Healthcare). Sequence data were analyzed with Sequencher version 4.7 (GeneCodes, Ann Arbor, MI).

## Results

### Non-parametric linkage analysis

The results of the non-parametric linkage analysis and the association tests for all 88 EMDs and all candidate gene flanking microsatellites are shown in Appendix 2. The highest Z-means of 1.94, 2.01, and 2.08 were obtained for the markers *LIM2_1_107.53*, *LIM2_1_108.39*, and *FTL_1_109.84*, which are located closely to the candidate genes, *FTL* and *LIM2*, on CFA1 (Appendix 2). These markers were significantly associated with CAT. Thus, we genotyped 30 additional microsatellite markers located on CFA1 to verify linkage with CAT and to delimit the linked region on CFA1. After that, the highest Z-mean and LOD score of 3.58 and 1.29 were obtained for marker *ABGc006*, which is located about 14 Mb proximal of *LIM2_1_108.39* and *FTL_1_109.84* (Appendix 3). The chromosome-wide error probabilities for this linked marker were at 0.0002 and 0.007, and the genome-wide error probability of the Z-mean was at 0.004. The test statistics Z-mean and LOD score for *LIM2_1_107.53*, *LIM2_1_108.39*, and *FTL_1_109.84* also increased through the high marker density in the neighborhood and their high information content. Recombinations of CFA1 were mostly observed in the proximal part and only a few in the distal part, and thus, markers distal of *ABGc006* often shared the same haplotype with *ABGc006*. The maximum achievable Z-mean was 88.24, and the corresponding value for the LOD score was 9.97, indicating that the power of the analysis was high enough to detect genome-wide significant linkage. These maximum values can only be reached when all markers employed are fully informative for all affected relatives. We could not achieve such high values for the Z-mean and LOD score in our analysis because we used multi-generation pedigrees, which means the information content among affected relatives decreased due to long pedigrees pathways among affected relatives in the families. The genome-wide significantly linked region on CFA1 extended between 95.105 and 96.397 Mb, and this region was delimited by *ABGc005* at 95.105 Mb and *FH3883* at 96.397 Mb.

A haplotype on CFA1 including the microsatellites *ABGc006*, *ABGc007*, *REN211B17*, and *C01.643* was significantly associated with primary cataracts at p=0.0133 (χ^2^=20.85). The markers delimiting this haplotype were located from 96.07 to 102.041 Mb and spanned the linked region and the regions ~4 Mb distal of the linked region. The haplotypes containing the alleles ‘264–296–159–218’, ‘266–304–155–220’, and ‘276–300–155–220’ were associated with higher incidences of posterior polar cataract whereas the haplotypes including the alleles ‘262–308–155–228’ and ‘276–304–155–220’ showed lower incidences of posterior polar cataract. Further haplotypes had very low frequencies (<1%) and were not meaningful for association.

The microsatellite marker for canine *SIX5* (*FH2598*), the third candidate gene on this chromosome that is located about 2.5 Mb distal of FTL, achieved a considerably lower, insignificant Z-mean and LOD score in the linkage analysis.

### Mutation analysis of candidate genes FTL and LIM2 on CFA1

We performed a mutation analysis for the *FTL* and *LIM2* genes due to the significant linkage of the flanking markers. The canine *FTL* gene (LOC477042) consists of four exons, which could also be affirmed through a canine EST (CN000212). These four exons are interrupted by three short introns. Although we sequenced the whole genomic sequence of the canine *FTL* gene including more than 300 bp upstream of the start codon and downstream of the stop codon, we did not find polymorphisms in the animals investigated here. We also sequenced all exons with their flanking intronic regions of the *LIM2* gene. This gene is located about two megabases (Mb) proximal of *FTL* on CFA1. The canine *LIM2* gene (LOC611737) consists of five exons of which the first exon is untranslated. In comparison to the human *LIM2* gene, exon 3 of the canine *LIM2* gene is 126 bp shorter. This result could be verified using cDNA analysis of the lens tissue of a Tibetan terrier. We did not find any exonic polymorphisms in the *LIM2* gene, but three single nucleotide polymorphisms (SNPs) in the non-coding regions were found. A significant association of these SNPs with primary cataracts in the EMD was not evident (Table 2).

**Table 2:** Entlebucher mountain dog *LIM2* polymorphism information.

**SNP**	**Location**	**PIC (%)**	**HET (%)**	**χ^2^ genotype**	**P genotype**	**χ^2^ allele**	**P allele**
LOC611737 g.4367G>A	Intron 2	35.5	58.6	1.67	0.43	1.20	0.27
LOC611737 g.4396A>G	Intron 2	20.3	14.7	3.67	0.16	4.32	0.04
LOC611737 g.6430T>G	3’UTR	19.9	14.3	3.50	0.17	4.13	0.04

Heterozygosity (HET), polymorphism information content (PIC), χ^2^-tests of the case-control analysis with their corresponding error probabilities (P) for the single nucleotide polymorphisms (SNPs) in the *LIM2* gene in the Entlebucher mountain dogs are presented.

## Discussion

We could show putative linkage for a genomic region on CFA1 with canine posterior polar cataract. This primary cataract formation is typical for EMDs, and a recessive major gene was shown to be involved in development of this condition. Genotyping of 31 microsatellites on CFA1 delimited the putatively linked region to about 1.3 Mb. In addition, a significantly associated haplotype including this linked region corroborated the results of the linkage analysis. Genome-wide and chromosome-wide linked markers supported this linked interval on CFA1. We sequenced the complete coding sequence and flanking intronic and untranslated regions of candidate genes, *LIM2* and *FTL*, on CFA1, but we could not find any polymorphism associated with the CAT phenotype in the EMD. We therefore ruled out the sequence investigated in these two genes for harboring the causative mutation for CAT. As the flanking marker (*FH2598*) of *SIX5*, which is also located on CFA1, is not included in the associated haplotype, we assumed that SIX5 is not involved in the pathogenesis of CAT in the EMD. To date, there are no other cataract candidate genes known in this putatively linked region, and we also could not find any further cataract candidate genes by searching this region in the current dog genome assembly 2.1. Further development and genotyping of SNPs for this putatively linked region and for the region significantly associated with the CAT phenotype is necessary to refine the haplotype shared by the CAT-affected dogs, which will enable the ability to identify associated genes and to screen these genes for CAT-causing mutations in the EMD.

## Appendix 1. Oligonucleotide primer sequences.

To access the data, click or select the words “Appendix 1.” This will initiate the download of a pdf archive that contains the file. Shown are PCR primers with their product size range and the annealing temperature (T_a_) for the amplification of markers flanking the canine cataract candidate genes.

## Appendix 2. Candidate gene flanking microsatellites in the Entlebucher mountain dog.

To access the data, click or select the words “Appendix 2.” This will initiate the download of a pdf archive that contains the file. Shown are non-parametric test statistics Z-mean and LOD score, their error probabilities (P_Z_, P_L_), χ^2^-tests for allele and genotype distribution of the case-control analysis, degrees of freedom (DF) and their corresponding error probabilities (P) for the candidate gene flanking microsatellites in the Entlebucher mountain dog.

## Appendix 3. Microsatellites on canine chromosome 1 (CFA1) in the Entlebucher mountain dog.

To access the data, click or select the words “Appendix 3.” This will initiate the download of a pdf archive that contains the file. Shown are non-parametric test statistics Z-mean and LOD score, their error probabilities (P_Z_, P_L_), χ^2^-tests for allele and genotype distributions of the case-control analysis with their degrees of freedom (DF), and corresponding error probabilities (P) for all microsatellites on canine chromosome 1 (CFA1) in the Entlebucher mountain dog.
